# MicroRNA and transcriptome analysis in periocular Sebaceous Gland Carcinoma

**DOI:** 10.1038/s41598-018-25900-z

**Published:** 2018-05-14

**Authors:** John C. Bladen, Jun Wang, Ajanthah Sangaralingam, Mariya Moosajee, Caroline Fitchett, Claude Chelala, Michele Beaconsfield, Edel A. O’Toole, Michael P. Philpott, Daniel G. Ezra

**Affiliations:** 1 Centre for Cell Biology and Cutaneous research, Blizard Institute, Barts and The London School of Medicine and Dentistry, Queen Mary University of London, London, UK; 2 Centre for Molecular Oncology, Barts Cancer Institute, Queen Mary University of London, London, UK; 3 Department of Ocular Biology and Therapeutics, UCL Institute of Ophthalmology, London, UK; 4 Eyelid Oncology, Moorfields Eye Hospital, London, UK

## Abstract

Sebaceous gland carcinoma (SGC) is a rare, but life-threatening condition with a predilection for the periocular region. Eyelid SGC can be broadly categorised into two subtypes, namely either nodular or pagetoid with the latter being more aggressive and requiring radical excision to save life. We have identified key altered microRNAs (miRNA) involved in SGC shared by both subtypes, hsa-miR-34a-5p and hsa-miR-16-5p. However, their gene targets *BCL2* and *MYC* were differentially expressed with both overexpressed in pagetoid but unchanged in nodular suggesting different modes of action of these two miRNAs on *BCL*/*MYC* expression. Hsa-miR-150p is nodular-specifically overexpressed, and its target *ZEB1* was significantly downregulated in nodular SGC suggesting a tumour suppressor role. Invasive pagetoid subtype demonstrated specific overexpression of hsa-miR-205 and downregulation of hsa-miR-199a. Correspondingly, miRNA gene targets, *EZH2* (by hsa-miR-205) and *CD44* (by hsa-miR-199a), were both overexpressed in pagetoid SGC. CD44 has been identified as a potential cancer stem cell marker in head and neck squamous cell carcinoma and its overexpression in pagetoid cells represents a novel treatment target. Aberrant miRNAs and their gene targets have been identified in both SGC subtypes, paving the way for better molecular understanding of these tumours and identifying new treatment targets.

## Introduction

Sebaceous gland carcinoma (SGC) is a rare, aggressive cancer that has a predilection for the periorbital region, perhaps due to the multitude of glands surrounding the globe, but can come from extraocular sites, albeit mainly within the head region^1^. Geographical variation is significant with the incidence around 0.65 per 100,000 in Canada versus China where it represents almost a third of the malignant eyelid workload and second to BCC in frequency^2,3^. In Japan, the rate of SGC equalled that of BCC in one study^4^. Risk factors for developing SGC include radiation exposure; for example external beam radiotherapy which used to be the mainstay of treatment for retinoblastoma^5^. More aggressive features include vascular and lymphatic invasion, orbital invasion, involvement of both upper and lower eyelids, poor differentiation, multicentric origin, diameter greater than 10 mm, infiltrative growth pattern and pagetoid invasion of the adjacent epithelium^6^. SGC can be broadly divided in to two subtypes with highly contrasting prognosis, namely: nodular and pagetoid^7^. The nodular form often presents as a discrete mass on the eyelid and is more amenable to curative surgical resection whereas the pagetoid (intraepitheial spread on histology) subtype presents as non-specific thickening or redness of the eye and often contains skip lesions that requires more mutilating surgery to achieve complete clearance^8^. Surgical excision is currently the only available treatment and this may require removal of a normal seeing eye (exenteration) to protect life, thus a better understanding of its molecular biology along with identification of alternative treatment modalities is needed^9,10^.

Little is known about the molecular factors involved in SGC tumorigenesis although recent targeted whole exome sequencing of periocular SGC has revealed mutations in *TP53* and *RB1* genes^11^. Four differentially expressed microRNA (miRNA) have been identified in SGC (hsa-miR-486-5p and -184 upregulated; hsa-miR-211 and -518d downregulated), however, these miRNAs were compared to sebaceous adenoma rather than normal tissue and no histological subtype analysis was made^12^. Furthermore, progression from sebaceous adenoma to carcinoma is unknown, unlike the transition from colonic adenoma to adenocarcinoma, confounding the discovery of real SGC tumorigenesis markers.

SGC prognosis is dependent on the subtype, with pagetoid conferring a poorer prognosis and nodular a better prognosis^8^. In this study we assess periocular SGC whole-miRNA profile normalised to eyelid tarsal plate, the presumed tissue of origin. We have identified aberrantly expressed miRNAs unique to pagetoid and nodular SGC along with shared aberrant miRNAs. Subsequently, a combination of *in-silico* miRNA target search and transcriptome profiling, as well as miRNA-target gene network analysis, was performed to highlight miRNA targets in both subtypes.

## Results and Discussion

Sebaceous gland carcinoma is a rare, but aggressive cancer with surgical excision as the only option for definitive treatment. In order to carry out complete excision a margin of normal tissue needs to be removed and verified using histological examination. Certain subtypes of SGC present with a local mass, termed nodular SGC and is more amenable to local resection (see Supplementary Fig. S1A and S1C), as a result confer a better prognosis. In contrast, diffuse lid swelling which demonstrates intraepithelial spread on histological and sometimes skip lesions do not demonstrate clear margins for wide local excision, thus more aggressive excision of the orbital content is required resulting in a poorer prognosis (see Supplementary Fig. S1B and S1D). However, little is known about the molecular biology of these tumours and the relationship between SGC miRNA profile, transcriptome and its cancer behaviour including the differences exhibited by the nodular and pagetoid subtypes.

### Common SGC miRNA in both nodular and pagetoid subtype with target gene expression

**Figure 1:**
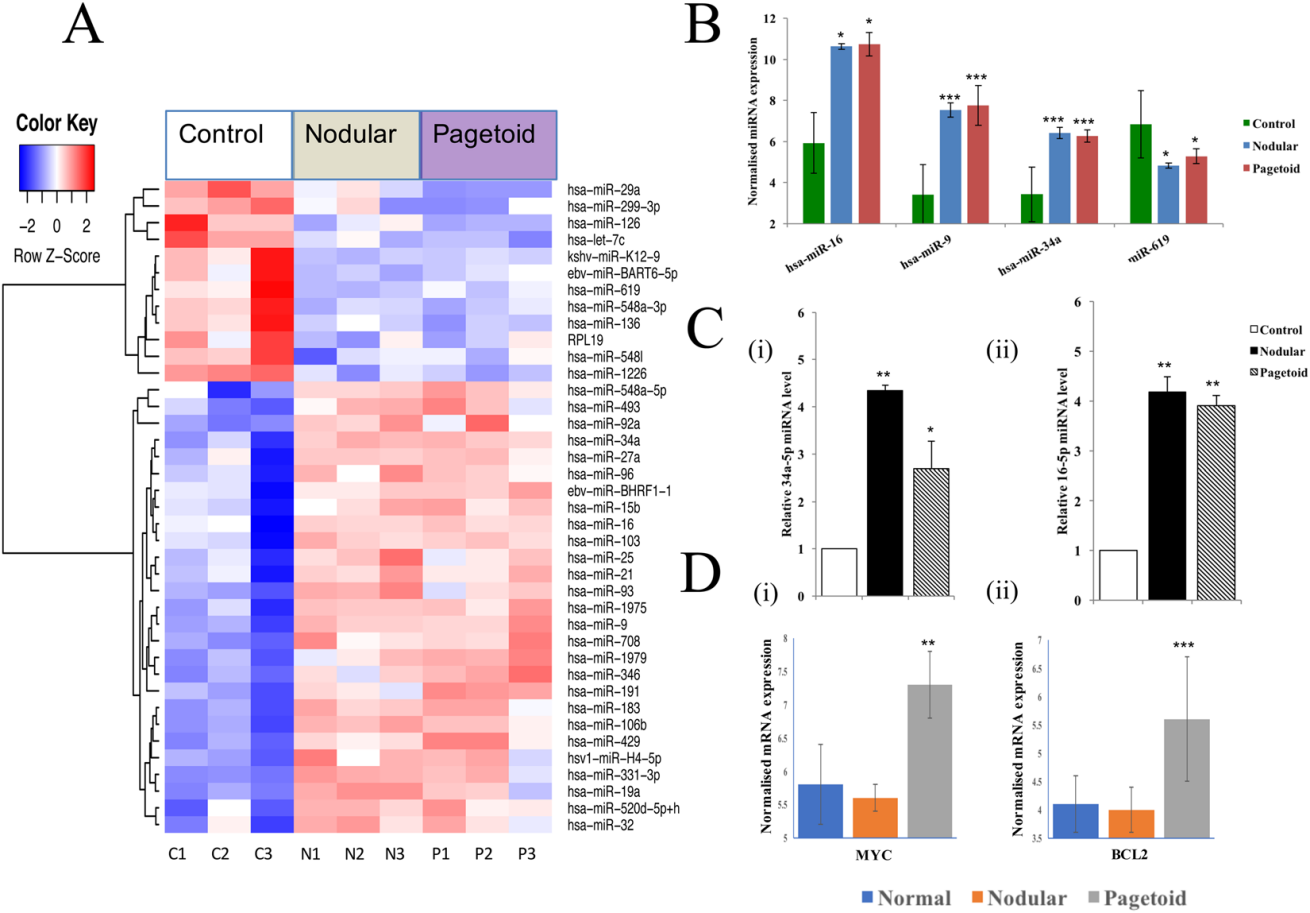
Shared microRNAs in nodular and pagetoid sebaceous gland carcinoma (SGC). (**A**) Thirty-nine significant differentially expressed microRNA shared between both subtypes using a *p* < 0.05 threshold. (**B**) Top 4 differentially expressed miRNAs present in both nodular and pagetoid SGC when compared to tarsal plate control. (**C**) MicroRNA verification of expression using Taqman RT-qPCR in SGC. Relative expression levels were determined for nodular and pagetoid SGC using Taqman RT-qPCR against normal eyelid tissue for miRNA (i) 34a-5p and (ii) 16-5p. (**D**) Expression of target genes in both nodular and pagetoid subtypes. Significance levels are shown as *P < 0.05, **P < 0.01, ***P < 0.001. Error bars represent mean +/− s.d.

Thirty-nine differentially expressed (DE) miRNA were common to both subtypes with the majority being upregulated (Fig. 1A). The top 4 DE miRNAs are shown in Fig. 1B (see Supplementary Table S1).

Hsa-miR-34a was significantly overexpressed in both subtypes compared to the control (Fig. 1B,C). It forms part of the TP53 suppressor network by modulating TP53 targets and forms a positive feedback loop via SIRT1^13^. Despite the common overexpression of hsa-miR-34a in both subtypes, the action of the miRNA on its target gene is different. For example, the target gene MYC was significantly overexpressed in the pagetoid subtype, but remaining unchanged in nodular (Fig. 1D). Interestingly in MYC-driven tumours, hsa-miR-34a improves cell survival based on its ability to reduce TP53 levels, in a MYC mediated and dependent fashion, hence helps the cancer cells to survive^14^. As *MYC* is over-expressed in the pagetoid subtype, our results suggest that hsa-miR-34a may play a tumour activating and enhancing role, but not the case in nodular SGC where the expression of *MYC* was unchanged compared to normal control (Fig. 1D). Furthermore, there is a known synergistic action between BCL2, also a target of hsa-miR-34a, and MYC in oncogenesis and this appears to be occurring in the pagetoid form only where both were significantly overexpressed (Fig. 1D)^15^. Hsa-miR-16 also targets BCL2 and was similarly overexpressed in both subtypes (Fig. 1B,C). Moreover, hsa-miR-16 has been shown to suppress both BCL2 and FOXO1, the latter being under-expressed in nodular SGC only^16^. It may therefore be acting in a similar opposing fashion as hsa-miR-34 and behave with a different mode of action according to the MYC and BCL2 status of the two subtypes. Hsa-mir-9, a known tumour suppressor, is overexpressed in both subtypes, and together with hsa-miR-619 (downregulated in both subtypes), targets MDGA2, a tumour suppressor gene which is highlighted in our common SGC miRNA-target gene network shown in Supplementary Fig. S2^17,18^. Both hsa-miR-126 and -9 target chemokine receptor CXCR4 which plays a role in metastatic spread^19,20^.

### Nodular SGC specific microRNA with target gene expression

**Figure 2:**
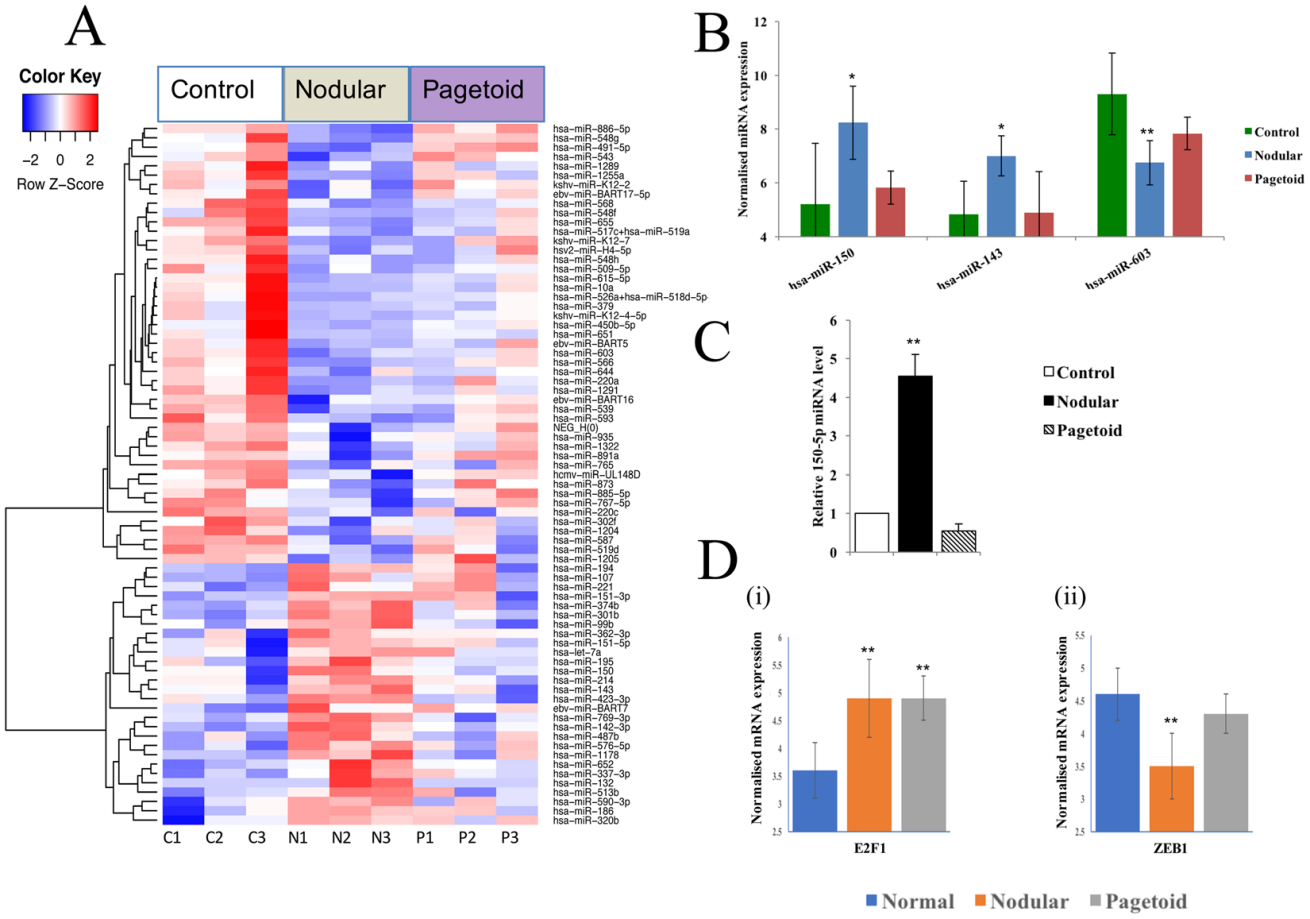
Nodular sebaceous gland carcinoma specific microRNAs. (**A**) Seventy-five significant differentially expressed microRNA unique to nodular SGC using a *p* < 0.05 threshold. (**B**) Top 3 differentially expressed miRNAs present in nodular SGC only when compared to tarsal plate control. Pagetoid expression shown to demonstrate non-significant change within the subtype. (**C**) MicroRNA verification of 150-5p expression using Taqman RT-qPCR in SGC. Relative expression levels were determined for nodular and pagetoid SGC using Taqman RT-qPCR against normal eyelid tissue for miRNA 150-5p. (**D**) Expression of target genes in nodular and pagetoid subtype for hsa-miR-150-5p. Significance levels are shown as *P < 0.05, **P < 0.01, ***P < 0.001. Error bars represent mean +/− s.d.

Seventy-five DE genes were found to be unique to nodular SGC compared to control and pagetoid (Fig. 2A). The majority of these (63%) were downregulated and the top 3 nodular specific miRNA are shown in Fig. 2B (see Supplementary Table S1).

Hsa-miR-150 was the most overexpressed miRNA unique to nodular SGC, with more than an 8-fold change (Fig. 2B,C) compared to control. Tumour suppressor action of hsa-miR-150 has been noted in epithelial ovarian cancer and shown to reduce invasion and metastasis by suppressing the transcriptional repressor *ZEB1* which was significantly downregulated in nodular SGC (Fig. 2D)^21^. Hsa-miR-143 was also found significantly upregulated specifically to the nodular subtype (Fig. 2B) and known for its tumour suppressive action^22^. It is likely to be acting through suppression of *BCL2*, which is also targeted by the aforementioned hsa-miR-16 and -34a, helping to prevent cancer progression. In contrast, *BCL2* is significantly overexpressed in the pagetoid subtype and as an important inhibitor of apoptosis, confers an advantage to the pagetoid subtype, however, BCL2 also represents a potential novel treatment target.^23^ Oncomir hsa-miR-603 was found to be much more downregulated in nodular than in pagetoid subtype (Fig. 2B) and we propose it is attempting to regulate of *E2F1* in a negatively correlated fashion which may contribute to nodular SGC less aggressive behaviour (Supplementary Fig. S3)^24,25^. A miRNA-target gene network of the proposed regulatory interactions seen in nodular SGC is shown in Supplementary Fig. S3. The MAPK/ERK pathway is intimately linked to the altered miRNAs seen in nodular SGC and it is possible that these miRNAs are acting as tumour suppressors to inhibit this pathway^26,27^. Moreover, synthetic miR-143 has been shown to silence KRAS signalling including its effector signalling molecules AKT and ERK (MAPK1).^28^ Furthermore, high expression of hsa-miR-150 specifically occurs in cancer patients and has the potential to be a biomarker of disease^29^.

### Pagetoid specific microRNA with target gene expression

**Figure 3:**
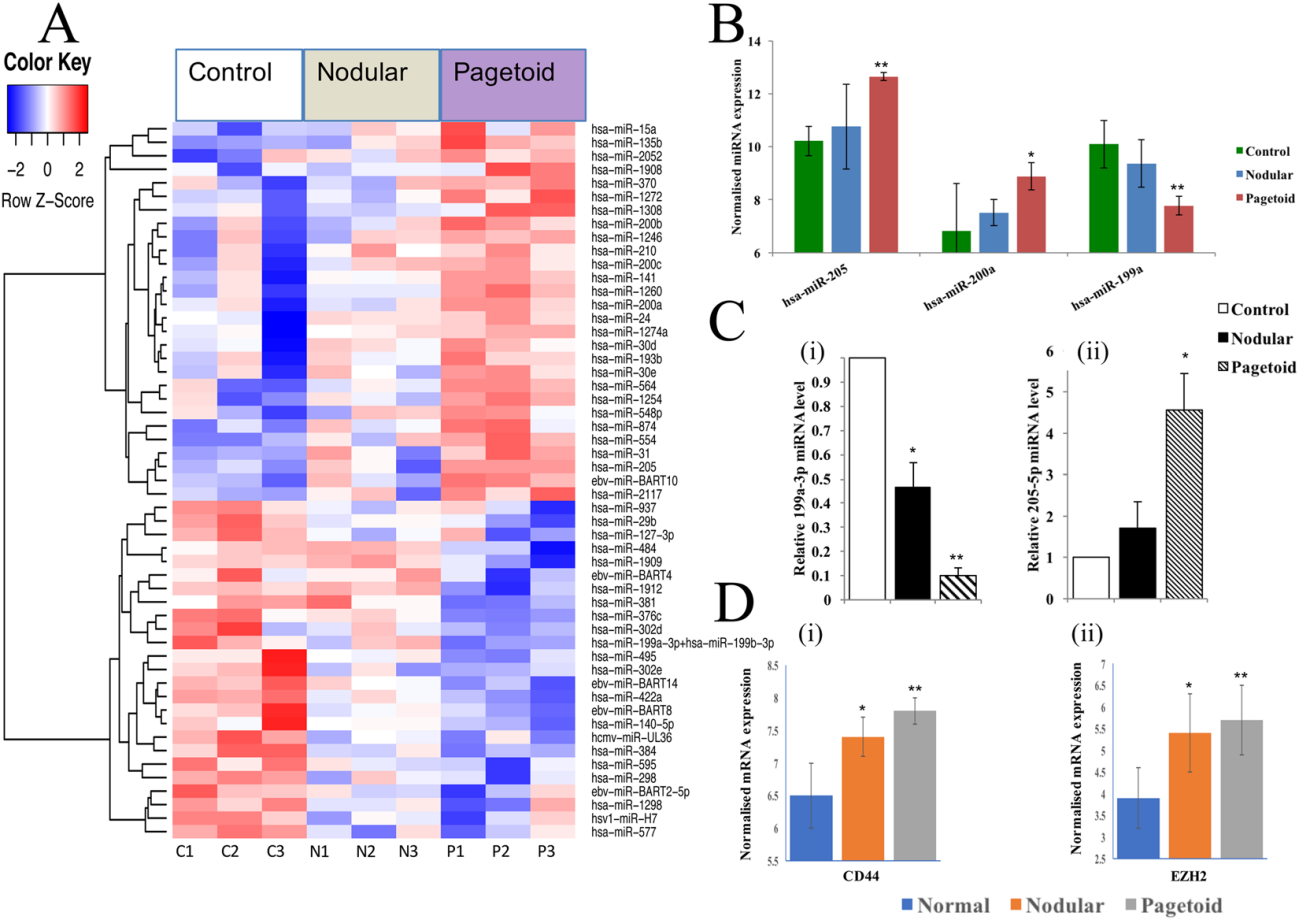
Pagetoid sebaceous gland carcinoma specific microRNAs. (**A**) Fifty-three significant differentially expressed microRNAs unique to nodular SGC using a p < 0.05 threshold. (**B**) Top 3 differentially expressed genes present in pagetoid SGC only when compared to tarsal plate. Nodular expression shown to demonstrate non-significant change within the subtype. (**C**) MicroRNA expression using Taqman RT-qPCR in SGC. Relative expression levels were determined for nodular and pagetoid SGC using Taqman RT-qPCR against normal eyelid tissue for miRNA (i) 199a-3p, (ii) 205-5p. (**D**) Expression of target genes in nodular and pagetoid subtype for hsa-miR-199a-3p and hsa-miR-205-5p. Significance levels are shown as *P < 0.05, **P < 0.01, ***P < 0.001. Error bars represent mean +/− s.d.

Pagetoid SGC expressed 53 specific, significantly DE genes (Fig. 3A). 47% of them were downregulated and the top 3 pagetoid specific genes are shown in Fig. 3B (see Supplementary Table S1).

Significantly increased level of hsa-miR-205 was found to be unique to pagetoid SGC and overexpression promotes VEGF invasion of ovarian cancer cells by targeting *EZR* and *LMNA* (Fig. 3B,C)^30^. Higher levels of hsa-miR-205 are also associated with adverse clinical outcome in bladder and biliary cancer through its action on ZEB1 by maintaining an epithelial phenotype^31,32^. Upregulation of hsa-miR-205 also occurs in non-squamous cell lung cancer and directly represses PTEN (targeted by multiple pagetoid specific differentially expressed miRNAs, including hsa-miR-205 and hsa-miR-200a – see Supplementary Fig. S4) and PHLPP2 expression, which in turn activates the AKT/FOXO3a and AKT/mTOR pathways respectively^33^. Moreover, an interaction between hsa-miR-205 and overexpressed EZH2 occurs in renal cell carcinoma to control b-catenin, and indeed EZH2 was found to be significantly overexpressed in pagetoid SGC (Fig. 3D)^34^.

Hsa-miR-200a, also a pagetoid specific upregulated miRNA, is involved in EMT too, but works together with hsa-miR-141 (upregulated in pagetoid only) to target MAPK14 to enhance the oxidative stress tumour growth response in ovarian cancer and effectively behaving as oncomiRs (Fig. 3B and see Supplementary Fig. S4).^35^

Hsa-miR-199 acts as a tumour suppressor gene in several cancers including colorectal and thyroid cancer^36,37^. Indeed, it was found to be the most downregulated miRNA unique to pagetoid SGC (Fig. 3B,C). Hsa-miR-199 has been shown to target CD44 glycoprotein, an oncoprotein that aids in cell adhesion and migration, with increased CD44 expression contributing to the aggressive nature of tumours and represents a therapeutic target plus possible stem cell biomarker^38,39^. This inverse association has also been noted in hepatocellular carcinoma^40^. CD44 was found to be significantly overexpressed in pagetoid subtype, which supports the suppressing role of hsa-miR-199 on CD44 seen in this subtype (Fig. 3). A summary of a pagetoid SGC specific miRNA-target gene network is shown in Supplementary Fig. S4, with common aforementioned gene targets highlighted along with the p53 feedback pathway (MAPK and PTEN).

### MiRNA-target gene networks and integrative analysis of sebaceous gland carcinoma

Integrative genomic analysis has been shown to be a powerful tool in identifying cancer subtype-specific highly connected network genes and signatures, which could offer potential subtype-specific drug targets^41–43^. These kind of studies often benefit from the availability of multi-omics data sets, especially on the same patient samples, such as TCGA studies: for example, in ovary^44^, lung^45^ and oesophageal^46^. The integration of different types of molecular data along with known human signalling plus protein-protein interaction, miRNA-target gene networks can further point us to a set of genes or pathways frequently targeted by various types of alterations and the interplay between them. To further identify robust SGC subtype specific signatures, we will need to generate and curate more multi-omic data sets (such as whole exome sequencing, RNA sequencing, copy number aberration and methylation), however SGC samples are rare to obtain, making it difficult to generate such datasets. More genomic studies involving multiple national and international centres are therefore much desired.

### Development of computational models for SGC related miRNA prediction

To further validate the functional significance and disease causal potential for our identified shared and specific miRNAs, curated, known miRNA-disease associations were queried for these miRNAs using HMDD v2.0^47^, MiRCancer^48^ and MiR2Disease^49^. These databases collect and record miRNA-disease associations through text mining in literature, manual confirmation, and experimentally supported evidence. Out of all 167 significantly differentially expressed miRNAs in SGC compared to control, ~100 (60%) were reported to be associated with at least one human disease with experiment-supported evidence based on HMDDv2.0. Similar percentages of miRNAs were also reported to have human disease and/or cancer associations based on MiRCancer and MiR2Disease, 60% and 48%, respectively. Thus, more than half of our SGC related miRNAs have been approved experimentally to be involved in other diseases and cancers.

Currently, there are little known, validated or predicted associations between miRNAs and SGC. Thus, it would be interesting to develop a computational framework for their association prediction, in addition to our Nanostring experimental data. This framework often requires first the prediction of miRNA functional similarity, disease semantic similarity, and the validated miRNA-disease associations. Similarity matrices are then integrated with Gaussian kernel similarity to produce the integrated similarity matrices for both miRNAs and diseases. Based on the assumption that functionally similar miRNAs tend to be associated with similar diseases, we can then identity and prioritise potential miRNA-SGC associations using graph-based learning (such as LRSSLMDA^50^ and PBMDA^51^), singular value thresholding algorithm (for example MCMDA^52^), or a within and between score model (for example WBSMDA^53^). Nevertheless, more studies are still needed to understand this rare and aggressive disease to improve the disease similarity prediction within this computational framework.

## Conclusion

We have identified novel aberrant miRNAs in SGC and related them to specific subtypes of SGC with different tumour behaviour to infer their role in carcinogenesis. Concomitantly, we assessed the transcriptome to explore the differential expression of predicted key target genes by these aberrant miRNAs, with the integration of miRNA-target gene networks associated with SGC and its subtypes. Further work is needed to understand the interplay of these novel miRNA in SGC tumour progression and their potential as treatment targets in the future.

## Methods

### Patients and tumours

This study included eight SGC samples from archival tissue housed at Moorfields Biobank and obtained both institutional (local) review board approval and national research ethics committee approval: Moorfields Eye Hospital Biobank internal research ethics committee agreement (reference: 10/H0106/57-2012ETR28), sponsor approval by Queen Mary University of London (number: 008621GM) and overall approval from the heath research authority, national research ethics service, Committee North West - Greater Manchester South (national research ethics committee number 14/NW/1080). As a result, all methods were carried out in accordance with relevant guidelines and regulations. In addition, all experimental protocols were approved by the aforementioned institutional and national ethical licensing committees. Four samples were pagetoid, 4 were nodular and compared to 4 normal tarsal plate as controls (see Supplementary Table S2). Six SGC were analysed for miRNA and 8 for messenger (mRNA) whilst being compared to tarsal plate control. Informed consent was taken for each patient for study publication and publication of identifying images in an online open access publication.

### Micro and messenger RNA extraction

RNeasy® FFPE kit from Qiagen® was used to extract both messenger and microRNA.

A Qubit® 2.0 Fluorometer (invitrogen™ by Life technologies™) and an Agilent 2100 bioanalyzer instrument (Agilent technologies, Inc) was used to determine concentration and integrity of messenger RNA (mRNA).

### Nanostring® nCounter®v2 microRNA expression assay and bioinformatics pipeline

Hybridisation of 800 unique oligonucleotide miRNA tags based on the miRBase version 18 reaction was performed. Raw miRNA expression and normalised data were first generated from Nanostring nCounter Digital Analyzer. Filtered data from the Nanostring platform were log2 transformed and further normalised using the quantile normalisation method in R (http://www.r-project.org/). Differential expression (DE) analyses were performed using the limma R package^54^. Pairwise comparisons of nodular versus control and pagetoid versus control were conducted to identify common and unique DE miRNA to the two subtypes using *p* < 0.05 (see Supplementary Table S1).

### Affymetrix™GeneChip® Human Gene 2.0 messenger RNA expression array and pipeline

Twelve samples were analysed (4 pagetoid, 4 nodular and 4 control) covering 47,000 transcripts. Expression data was normalised using the GC-robust multiarray average^55^. DE genes were identified using the threshold of p < 0.01 with limma R (see Supplementary Table S1). Data has been deposited to the Gene Expression Omnibus under the accession of GSE101476 (www.ncbi.nlm.nih.gov/geo).

### Real-time quantitative reverse transcriptase–PCR (rt-qPCR) for microRNA validation

A library of cDNA from isolated miRNA was made using TaqMan® Advanced miRNA cDNA synthesis kit. Customised oligonucleotides for micoRNA were designed (see Supplementary Table S3) and hsa-miR-26a-5p was chosen as the control (see Supplementary Table S4). A minimum of three replicates for each reaction and relative transcript expression was calculated by the 2^−ΔΔCt^ method and miRNA levels of each gene were normalised to the geometric mean of hsa-miR-26a-5p.

### *In silico* miRNA-target gene network

For selected top differentially expressed miRNAs, their targeted genes were identified in silico using miRNA target prediction databases (www.mirbase.org; www.mirdb.org; www.mirtarbase.mbc.nctu.edu.tw)^56–58^. The differential expression patterns of these target genes were then explored based on our mRNA expression data. The miRNA-target gene networks were further constructed using Cytoscape v3.3.0 (http://www.cytoscape.org/), with various shapes and colours indicating different attributes of miRNAs and genes.

### Accession codes

Data has been deposited to the Gene Expression Omnibus under the accession of GSE101476 (www.ncbi.nlm.nih.gov/geo).

## Electronic supplementary material

Supplementary Figure S1–4 and Table S2-S4

Supplementary Table S1
