# Supplementary material for: MicroRNA and transcriptome analysis in periocular Sebaceous Gland Carcinoma
Source: Sci Rep. 2018 May 14;8:7531. doi: 10.1038/s41598-018-25900-z (PMC5951834; doi:10.1038/s41598-018-25900-z)
Supplement: Supplementary file 1 — Supplementary Figure S1–4 and Table S2-S4 [file 41598_2018_25900_MOESM1_ESM.pdf]

Scientific Reports – Supplementary Figures and Tables

MicroRNA and transcriptome analysis in periocular Sebaceous Gland Carcinoma

John C. Bladen[1,3,4,*,†], Jun Wang[2,†], Ajanthah Sangaralingam[2], Mariya Moosajee[3], Caroline Fitchett[3], Claude Chelala[2], Michele Beaconsfield[4], Edel A. O'Toole[1], Michael P. Philpott[1], Daniel G. Ezra[3, 4]

[1] Centre for Cell Biology and Cutaneous research, Blizard Institute, Barts and The London School of Medicine and Dentistry, Queen Mary University of London, London, UK

[2] Centre for Molecular Oncology, Barts Cancer Institute, Queen Mary University of London, London, UK

[3] Department of Ocular Biology and Therapeutics, UCL Institute of Ophthalmology, London, UK

[4] Eyelid Oncology, Moorfields Eye Hospital, London, UK

*Correspondence and requests for materials should be addressed to J.C.B (email: j.bladen@qmul.ac.uk)

[†] These authors made an equal contribution

Supplementary figures and legends

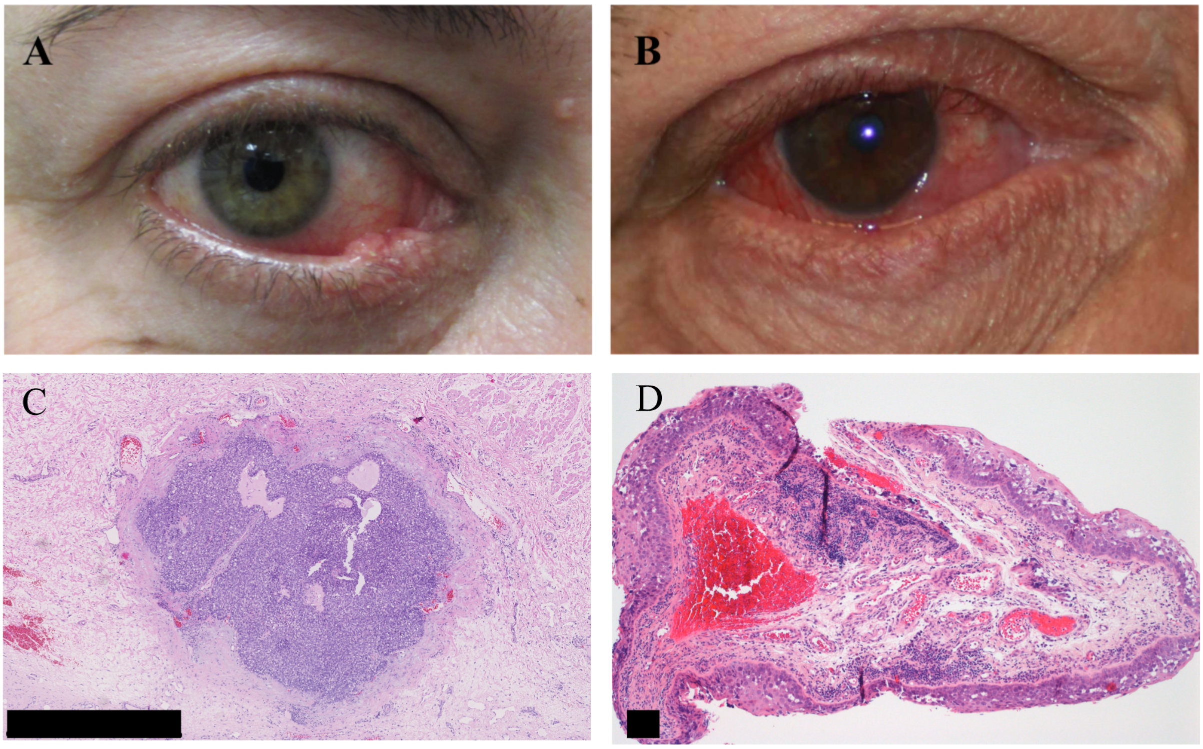

Supplementary Figure S1. Clinical and pathological features of sebaceous gland carcinoma (SGC). (A) Nodular SGC of the right lower eyelid (B) Pagetoid SGC involving bulbar and tarsal conjunctiva of the upper and lower eyelid (C) H+E of nodular SGC demonstrating localised collection of frothy, acinar like arranged cells with intracytoplasmic fat. (D) H+E of pagetoid SGC demonstrating scattered invasive tumour within the conjunctiva of the eyelid intermixed with more normal looking conjunctival cells. Scale bar 1mm.

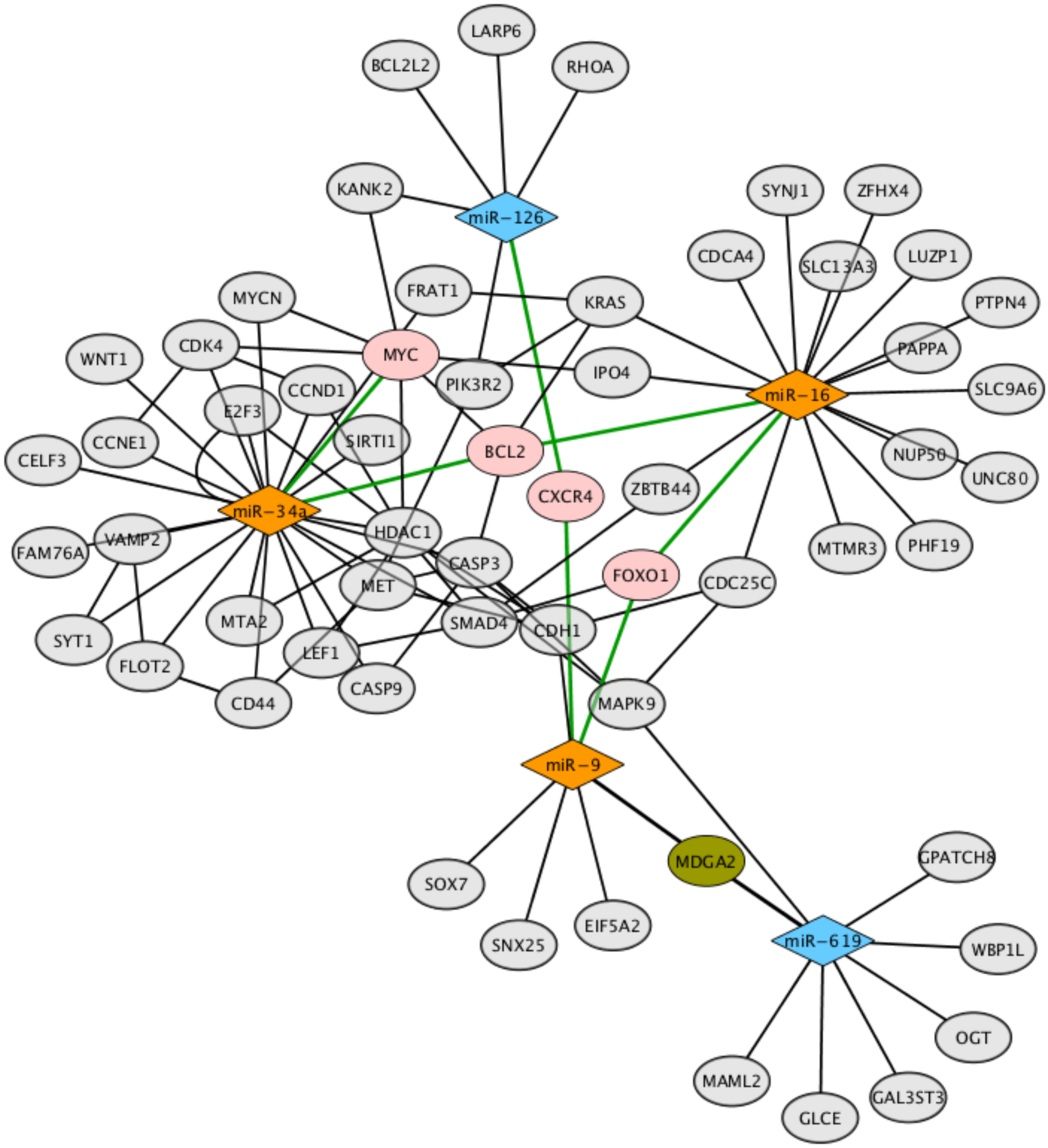

Supplementary Figure S2. MicroRNA-target gene network for top DE miRNAs common to both subtypes of sebaceous gland carcinoma. Similarly altered miRNA in both subtypes are shown, however, their target genes are differentially affected. Target genes are represented with an oval shape and a diamond shape was used for miRNAs. Orange color highlights upregulation and blue highlights downregulation for miRNAs. Pink nodes mean that the expression patterns of genes were different between nodular and pagetoid subtypes. For example, BCL2 upregulated in pagetoid and downregulated in nodular; CXCR4 is upregulated

in pagetoid and no change in nodular; FOXO1 had no change in pagetoid and downregulated in nodular; MYC is upregulated in pagetoid and downregulated in nodular. Olive node means the gene was not differentially expressed or the expression was not available or filtered out during quality control. Green edges indicate the interaction between miRNAs and genes are in pink. Interactions between miRNAs and genes in olive were marked with thick black lines.

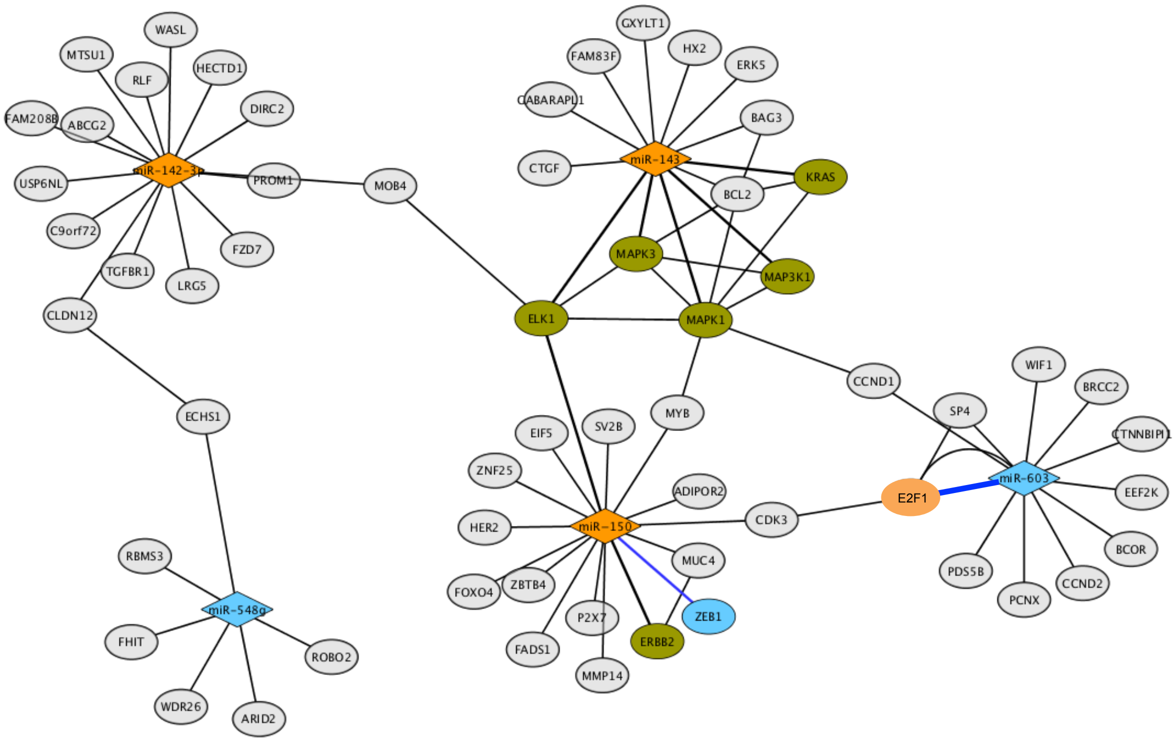

Supplementary Figure S3. Nodular specific microRNA-target gene network. Top altered microRNA are highlighted with their gene targets. Target genes are represented with an oval shape and a diamond shape used for miRNAs. Orange color highlights upregulation and blue highlights downregulation for miRNAs. Olive node means the gene was not differentially expressed or the expression was not available or filtered out during quality control. Red line indicates detected positive correlation and blue line indicates a negative correlation between miRNA and targeted genes. Interactions between miRNAs and genes in olive were marked with thick black lines. Oliver genes connected with miR-143 are all involved in MAPK/ERK pathway.

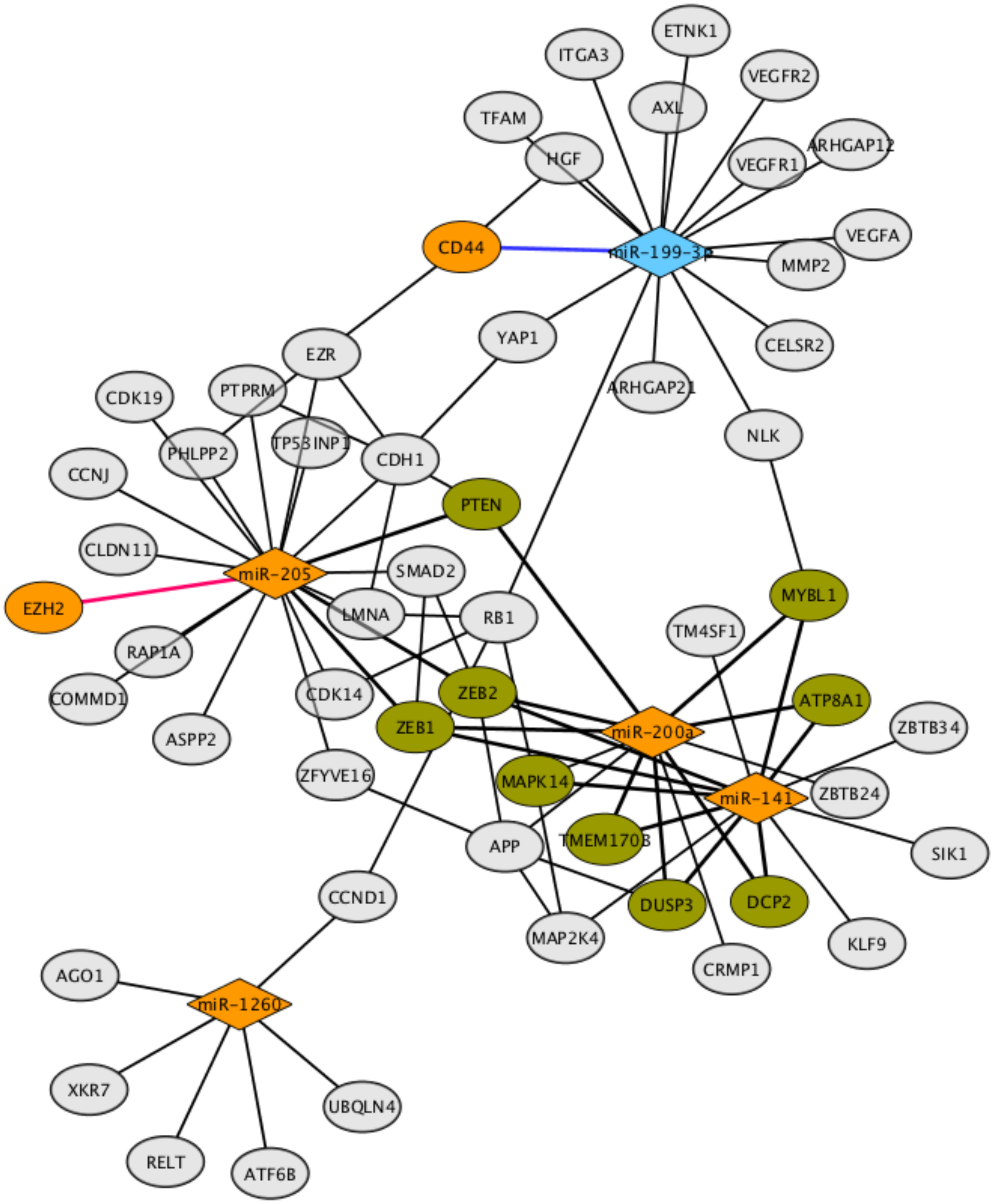

Supplementary Figure S4. Pagetoid specific microRNA-target gene network. Top altered microRNA are highlighted with their gene targets. Target genes are represented with an oval shape and a diamond shape used for miRNAs. Orange color highlights upregulation and blue highlights downregulation for miRNAs. Olive node means the gene was not differentially

expressed or the expression was not available or filtered out during quality control. Red line indicates detected positive correlation and blue line indicates a negative correlation between miRNA and targeted genes. Interactions between miRNAs and genes in olive were marked with thick black lines. Many olive genes in this network seemed to be targeted by multiple miRNAs, e.g., miR-200a, miR141 and miR-205.

Tables

Sample	Age	Gender	Side	Subtype	miRNA study	mRNA study
**P1**	70	F	RE	Pagetoid	Y	Y
**P2**	71	F	LUL	Pagetoid	Y	Y
**P3**	64	F	RE	Pagetoid	Y	Y
**P4**	66	M	RE	Pagetoid		Y
**Sample**	**Age**	**Gender**	**Side**	**Subtype**		
**N1**	66	F	RUL	Nodular	Y	Y
**N2**	72	F	LLL	Nodular	Y	Y
**N3**	78	M	LUL	Nodular	Y	Y
**N4**	65	F	LUL	Nodular		Y
**Sample**	**Age**	**Gender**	**Side**	**Subtype**		
**C1**	73	F	LUL		Y	Y
**C2**	67	F	LUL		Y	Y
**C3**	77	M	LUL		Y	Y
**C4**	63	F	RUL			Y

Supplementary Table S2. Demographics of the 8 sebaceous gland carcinoma and 4 tarsal plate control in the study. F, female; M, male; RE, right eye; UL, upper eyelid; LL, lower eyelid.

Mature miRNA	Accession number	TaqMan oligo
hsa-miR-16-5-p	MIMAT0000069	UAGCAGCACGUAAAUAUUGGCG
hsa-miR-34a-5p	MIMAT0000255	UGGCAGUGUCUUAGCUGGUUGU
hsa-miR-205-5p	MIMAT0000266	UCCUUCAUUCCACCGGAGUCUG
hsa-miR-150-5p	MIMAT0000451	UCUCCCAACCCUUGUACCAGUG
hsa-miR-199a-3p	MIMAT0000232	ACAGUAGUCUGCACAUUGGUUA
hsa-miR-26a-5p (control)	MIMAT0000082	UUCAAGUAAUCCAGGAUAGGCU

Supplementary Table 3 Primer design for microRNA. Customised primers for Taqman® real time quantitative PCR was used in conjunction with Invitrogen™ (Life technologies™). In addition, an endogenous control gene, from microRNA derived cDNA. MiRNA were chosen from Nanostring® nCounter® human v2 MicroRNA expression assay.

	Nodular vs. control		Pagetoid vs. control	
**MiRNA**	**Log2FC**	**Expression**	**Log2FC**	**Expression**
miR-361-5p	-1.3	5.0	0.05	4.7
miR-186-5p	1.9	4.0	1.2	4.0
miR-26a-5p	-0.33	11	-0.12	11
miR-191-5p	1.3	6.3	2.3	6.3
miR-451a	-0.40	8.7	0.16	8.7
miR-423-5p	0.84	5.4	0.3	5.4
miR-320a	-0.6	3.1	-0.4	3.1

Supplementary Table 4. Endogenous microRNA control selection. Listed are all possible endogenous controls for subsequent Taqman real time quantitative PCR with hsa-miR-26a-5p chosen as the control as it is highly expressed in both tissue subtypes and similarly activated.
